# Effect of Hydroxychloroquine in a Mouse Model of Abortion Caused by *Brucella abortus* Infection

**DOI:** 10.1155/tbed/8853857

**Published:** 2025-08-08

**Authors:** Haoran Liu, Zhirong Yan, Jingyu Wang, Xiaofeng Liu, Weiyu Zhang, Jun Liu, Qisheng Peng

**Affiliations:** ^1^State Key Laboratory for Diagnosis and Treatment of Severe Zoonotic Infectious Diseases, Key Laboratory for Zoonosis Research of the Ministry of Education, Institute of Zoonosis, and College of Veterinary Medicine, Jilin University, Changchun, Jilin, China; ^2^Clinical Laboratory, Tumor Hospital of Jilin Province, Changchun, Jilin, China; ^3^Jilin Provincial Center for Disease Control and Prevention, Institute of Microbiology Department, Changchun, Jilin, China; ^4^Changchun Veterinary Research Institute, Chinese Academy of Agricultural Sciences, Changchun, Jilin, China

**Keywords:** abortion, *Brucella*, hydroxychloroquine, inflammation, type IV secretion system

## Abstract

Hydroxychloroquine (HCQ) has showed beneficial effects on pregnancy outcomes in systemic erythaematosus lupus (SLE) by its anti-inflammatory and immunomodulatory properties. However, the role of HCQ in preventing abortion caused by infectious diseases remains unknown. In this study, we choose *B. abortus*-induced abortion of pregnant mice as a model to investigate the effect of HCQ in preventing abortion. HCQ decreases the pH value of endosomal *Brucella*-containing vacuole (eBCV), *B. abortus* type IV secretion system (T4SS) expression in trophoblasts and *Brucella* intracellular growth and cell death. Moreover, administration of HCQ decreases inflammation triggered by dead trophoblasts in a T4SS-independent manner. Mechanistically, this suppressed inflammatory response is due to inhibition of both NF-kB and mitogen-activated protein kinases (MAPKs) activation by HCQ. In the murine model of abortion, HCQ treatment enhanced pup viability through suppressing *B. abortus* growth within placenta and preventing placentitis. Our results suggest administration of HCQ could be an effective protection against abortion induced by *Brucella* infection, which imply that HCQ may be beneficial in treating abortion caused by infectious diseases.

## 1. Introduction

Brucellosis, which is caused by bacteria of the genus *Brucella*, is a neglected and a reemerging zoonosis with worldwide distribution. *Brucella* is facultatively intracellular bacteria that can infect, survive, and multiply in different cell types in vitro or in vivo. In the natural host, *Brucella* infection causes abortion of pregnant animals, which results in highly relevant economic losses for the animal industry [1, 2]. Placentitis and retained placenta in females are the main clinical signs and lesions [3]. However, there is currently no effective treatment to prevent brucellosis induced abortion [2].

Previous studies indicated that erythritol and other suitable energy sources available in the placenta are responsible for favoring *Brucella* multiplication in the placenta. The tropism of *Brucella* for the maternal–fetal barrier favors fetal infection [1, 4, 5]. During the course of infection, *Brucella* prefers to infect trophoblasts in pregnant animals. The highly *Brucella*-infected trophoblasts in placenta cause cell death, releasing damage-associated molecular patterns (DAMPs) into the placenta to activate placental macrophages to induce acute inflammation [6, 7]. Neutralizing high mobility group box 1(HMGB1), TNFα, or administration of progesterone or endoplasmic reticulum (ER) stress inhibitors, TUDCA, decrease inflammation, and increase pup viability in the *Brucella*-infected pregnant mice [7–10]. Although these findings suggest that suppression of inflammation in the placenta plays an important role in protecting abortion, pup viability of infected mice did not exceed 35% using these treatments compared to no treatment. The lower ratio of pup viability maybe due to capacity limitation of these treatment in inhibiting inflammation. Our goal is to find a drug that can decrease the ratio of abortion through decreasing inflammation.

*Brucella* type IV secretion system (T4SS), which is encoded by the *virB* operon, was shown to be required for virulence in a murine model of chronic brucellosis and bacterial intracellular replication. Deletion of genes within the *virB* operon rendered *Brucella* unable to convert endosomal *Brucella*-containing vacuole (eBCV) into replicative *Brucella*-containing vacuole (rBCV) and multiplication intracellularly [11]. If trophoblasts are infected with T4SS deficient *Brucella*, lower bacterial loads do not lead to cell necrosis, which can not cause acute inflammation. In line with low inflammatory response, pregnant mice infected by T4SS deficient *Brucella* do not induce abortion [12, 13]. The acidic environment in eBCV mediates the expression of T4SS [14]. In the initial hours following infection, initial BCV briefly fuzes with the late lysosome to create eBCV in an acidic environment. Inhibition of lysosomal acidification prevents the induction of the T4SS expression [11].

HCQ (Hydroxychloroquine), which is derived from quinacrine, has been utilized as an antimalarial drug since 1955. With the discovery of new properties, HCQ was often utilized as an immunomodulatory and anti-inflammatory drug for the treatment of autoimmune diseases, such as systemic erythaematosus lupus (SLE) and rheumatoid arthritis (RA). HCQ is also proven to be safe and effective in treating the chronic endothelial dysfunction of pregnant women with SLE. In a recent study of 251 SLE patients with 263 pregnancies, HCQ was associated with a lower risk of adverse pregnancy outcomes [15–19]. Additionally, HCQ is commonly used as an agent to raise the pH of lysosomes [20]. However, the role of HCQ in preventing abortion caused by infectious diseases remains unknown. Here, we choose *B. abortus* infection-induced abortion as a model to investigate whether administration of HCQ provides effective abortion protection. This study will broaden the understanding of HCQ regulation in abortion.

## 2. Materials and Methods

The details of this section are are available in the online-only Supporting Information.

### 2.1. Ethics Statement

Experimental C57BL/6 mice were purchased from the Jillin University. The study is approved by the Animal Experimental Ethical Committee of Jilin University (Agreement Number: KT202408159).

### 2.2. Construction of *B. abortus* Expressing HA-VceC

To create a *B. abortus* 2308 strain in which HA-VceC is overexpressed, we first designed the VceC forward primer containing HA gene (TGAATTC ATGTACCCATACGATGTTCCAGATTACGCGCCGTTTCAGCGGC), then designed the reverse primer (TTCTAGA CTAGCGGGTTTCTCCCTTG). We obtained the whole VceC gene containing HA tag through PCR, which was cloned into pBBR1MCS-2 vector with Xho1and EcoRI restriction enzymes sites. The plasmid was tested by DNA sequencing. And then this vector was introduced into the *B. abortus* by electroporation. To analyze HA-VceC expression in *Brucella*, *B. abortus* containing pBBR1MCS-2-HA-VceC (including KanR) was cultured in tryptic soy broth (TSB) to an OD600 value of 0.25. The HA-VceC expression was determined by Western blotting through using mouse anti-HA antibody (CST, Cat##65738).

### 2.3. Exposure of *B. abortus* to Environmental Change

To examine the effect of growth phase on *virB*11 expression, *B. abortus* was grown to early exponential phase in TSB or modified minimal E medium with glucose in the presence or absence of HCQ (Sigma–Aldrich, Cat#509272) (1 μg/mL) in shaker [21, 22].

### 2.4. Quantitation of *virB*11 or VjbR Gene Expression

The analysis of *virB* or VjbR gene expression during infection was performed using quantitative real-time PCR (RT-PCR). The indicated primers were used for RT-PCR of either *virB*11 (*virB*11-f [5′-TGACTTTCGAGCGGCTGG-3′] and *virB11*-*r* [5′-GCACGACGACATCCACCA-3′]) or VjbR (VjbR -f [5′-GCCCTCCTGCCTGCCTGAAA-3′] and VjbR -*r* [5′- GTCACCACCGTCCCGGGCTTC -3′]) mRNAs. Mean n-fold expression levels of cDNA from three independent biological replicates, each measured in duplicate, were normalized to genomic DNA (gDNA) levels and calibrated according to the threshold cycle method [23].

### 2.5. Measurement of eBCV pH

pH value of eBCV was measured following previously described procedures [24]. Briefly, HPT-8 cells were infected with carboxyfluorescein (CF)-Rho -labeled *Brucella* (MOI = 100) for 1 h. Cells were then washed with PBS twice and add gentamicin (30 μg/mL) in complete DMEM medium (Gibco, Cat#11965092) to kill extracellular bacteria. And then the infected cells were incubated for 1 h in a various buffer of defined pH (3.5, 4, 5, 6, 7, and 8), respectively. A calibration curve of the CF/Rho emission ratio versus pH by fluorescence microscopy was obtained at the end of each experiment. For HCQ administration, HCQ (1 μg/mL) was added into medium during the stage of eBCV formation (between 0 and 8 h post infection). Based on calibration curve, pH value of eBCV was calculated through the CF/Rho ratio.

### 2.6. BMDMs Stimulation With Conditioned Media(CM)

Mice trophoblasts were infected with WT or *virB*11 deficient *B. abortus* in the presence or absence of HCQ (1μg/mL) for 48 h, or cells were infected with *B. abortus* for 7 h, and then add HCQ for another 41 h. The infected CM from above infected cells were harvested and sterilized by filtration through 0.22 μm nitrocellulose filters. CM were diluted 1/4 in complete medium for stimulating BMDMs. After 24 h stimulation, the supernatants were collected for analyzing cytokines. To calculate the specific secretion of each factor, levels already present in CM from cells were subtracted from levels measured after stimulation [7].

### 2.7. Statistical Analysis

Data are reported as mean ± S.D. Comparisons between two groups were analyzed by unpaired Student's *t* test. A *p*-value < 0.05 was considered significant. One-way ANOVA followed by Bonferroni correction is used for analyzing data.

## 3. Results

### 3.1. Effect of HCQ on eBCV Acidification

**Figure 1:**
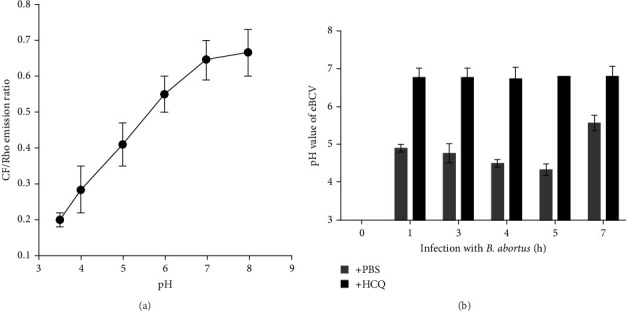
Effect of HCQ on eBCV acidification. *B. abortus* was labeled with fluorescent probes NHS-Rho and NHS-CF. (A) The calibration curve for the calculation of pH value of eBCV based on the CF/Rho emission ratio in vitro. (B) HPT-8 cells were infected with the labeled bacteria as indicated times in the presence of PBS or HCQ (1 μg/mL), and eBCV pH was measured. Data represent means ± SD.

Upon entry into host cells, *Brucella* reside within a membrane-bound compartment that interact with early and late endosomes and lysosomes to become an acidified eBCV (between 0 and 8 h post infection) [11]. As HCQ can accumulate inside cells and alkalinizes cells phagolysosome, causing an increase of pH value [20]. We investigated the effect of HCQ on eBCV acidification. First, HPT-8 cells were infected with *Brucella* that was labeled with fluorescent probes in a buffer of defined pH. The calibration curve was established through calculation of pH from the CF/Rho emission ratio (Figure 1A). As expected, eBCV acidification was suppressed by HCQ compared to PBS treatment (Figure 1B).

### 3.2. HCQ Inhibits the Expression of Intracellular *Brucella* T4SS by Raising Early pH of eBCV

**Figure 2:**
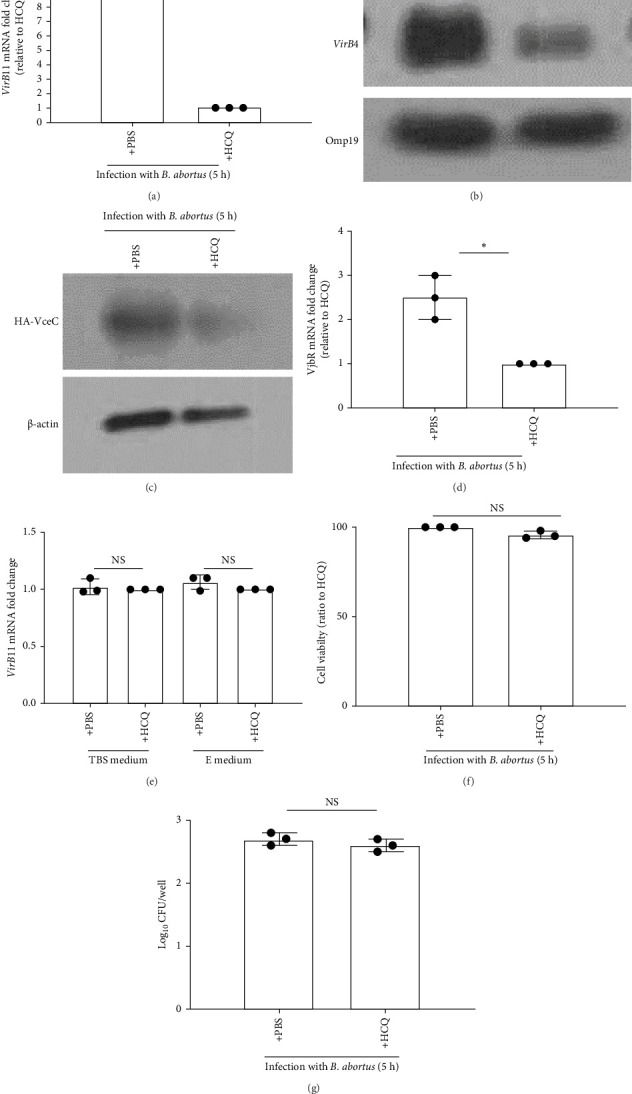
HCQ inhibits the expression of intracellular *Brucella* T4SS by raising early pH of eBCV. HPT-8 cells were infected with *B. abortus* (MOI:100) in the presence of PBS or HCQ (1 μg/mL) for 5 h. (A) The expression of *virB*11 within HPT-8 cells is analyzed by qRT-PCR. Data are showed as fold changes compared to HCQ treatment. (B) The expression of *virB*4 within HPT-8 cells is analyzed by western blotting. Bacterial Omp19 was designed as loading control. (C) HPT-8 cells were infected with *B. abortus* containing HA-Vcec vector in the presence of HCQ (1 μg/mL) or PBS for 5 h. Cytosolic Vcec production in cells was analyzed by western blotting with anti-HA. β-actin expression was designed as a loading control. (D) The expression of VjbR within HPT-8 cells was analyzed by qRT-PCR. Data are showed as fold changes compared to HCQ treatment. (E) Bacteria were grown to early exponential phase in rich TSB or minimal medium pH 7.0 with glucose (E), and qRT-PCR analyzed *virB*11 expression in bacteria. Data are showed as fold changes compared to HCQ treatment. (F) The viability of HPT-8 cells was examined by MTT assay. Data are showed as fold changes compared to HCQ treatment. (G) The bacterial number within HPT-8 cells was measured by CFU assay. One-way ANOVA followed by Bonferroni correction is used for analyzing results. *p* values less than 0.05 is statistically significant (*⁣*^*∗*^, *p<*0.05). NS: no significance.

As the acidification of eBCV plays an essential role in inducing the expression of T4SS that is encoded by the *virB* operon [22, 25], and our data (Figure 1B) showed that eBCV acidification peaks at 5 h post infection, we selected the time point, 5 h post infection, to study the effect of HCQ in mediating the expression of intracellular bacterial T4SS. Our data demonstrated that *virB*11 expression was suppressed while HPT-8 cells were infected with *Brucella* in the presence of HCQ (Figure 2A). To confirm that HCQ can suppress T4SS expression, we also checked the effect of HCQ in mediating the expression of *virB4* (another gene of *virB* operon) (Figure 2B), effector VceC (Figure 2C), which is secreted into host cells through T4SS, and the mRNA transcription of VjbR (Figure 2D), which is activating transcription factor of T4SS. Consistent with *virB*11 expression, HCQ inhibited VecC and *virB4* expression and the levels of mRNA of VjbR. To exclude the possibility that the inhibitory effect of HCQ in T4SS expression is due to direct effect on *virB* expression of HCQ and the difference of intracellular bacterial growth and host cell viability. Our data showed that HCQ did not affect *virB* expression on cultured *Brucella* under normal conditions or conditions that favor expression of the *virB* operon (Figure 2E) [22]. HCQ treatment leads to the similar intracellular CFU numbers and host cell survival as nontreatment at 5 h post infection (Figures 2F,G). Collectively, these results show that T4SS expression of intracellular bacteria can be suppressed by HCQ through raising pH of eBCV.

### 3.3. HCQ Suppresses Growth of *B. abortus* Within Trophoblasts in T4SS Dependent Manner

**Figure 3:**
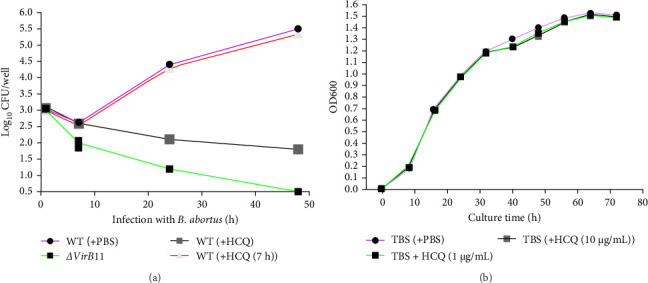
HCQ suppresses the multiplication of *B. abortus* within trophoblasts in T4SS dependent manner. (A) HPT-8 cells were infected with WT *B. abortus* (MOI = 100) in the presence of PBS, HCQ (1 μg/mL) or HCQ (1 μg/mL) treatment after 7 h of *Brucella* infection (HCQ (7 h) for the indicated times. The bacterial number within HPT-8 cells was measured by CFU assay. *ΔvirB*11 *B. abortus* infection (MOI = 100) was served as a positive control. (B) The WT *B. abortus* growth curve in TSB in the presence of 0, 1, or 10 μg/mL HCQ. Three independent experiments performed, and data represent means ± SD.

The T4SS expression was proven to be required for *Brucella* intracellular replication of in the phagocytic or nonphagocytic cells [11]. Next, we investigated whether the decrease of T4SS expression caused by HCQ mediates the bacterial growth within trophoblasts. In experiments, we introduced *ΔvirB*11 *B. abortus* group as positive control as deletion of *Brucella* T4SS can lead to significant decrease of intracellular bacterial growth [26]. HCQ treatment led to a constant decrease of *Brucella* intracellular growth compared to nontreatment (Figure 3A). Given the pH 4.5–5.0 of eBCV is essential for the induction of the *Brucella* T4SS [11], meanwhile, the pH value of eBCV exceeds 5.0 (almost 5.6) after 7 h of *Brucella* infection (Figure 1), we also investigated how HCQ regulates *Brucella* intracellular growth by introducing HCQ (7 h) treatment group (HCQ treatment after 7 h of *Brucella* infection). Interestingly, HCQ (7 h) did not affect intracellular replication of *B. abortus* (Figure 3A). These data suggested that the T4SS expression mediated by early acidic pH of eBCV regulates intracellular bacterial growth. To exclude the possibility that HCQ directly affects bacterial growth, we also checked the effect of HCQ on cultured bacterial survival. Evaluation of the growth of *Brucella* after treatment with HCQ (1 or 10 μg/mL) demonstrated that HCQ had no impact on *B. abortus* growth (Figure 3B). Taken together, these data suggest that HCQ inhibit bacterial intracellular multiplication in T4SS dependent manner.

### 3.4. HCQ Can Reduce Trophoblasts Death Through Inhibiting *Brucella* Intracellular Growth

**Figure 4:**
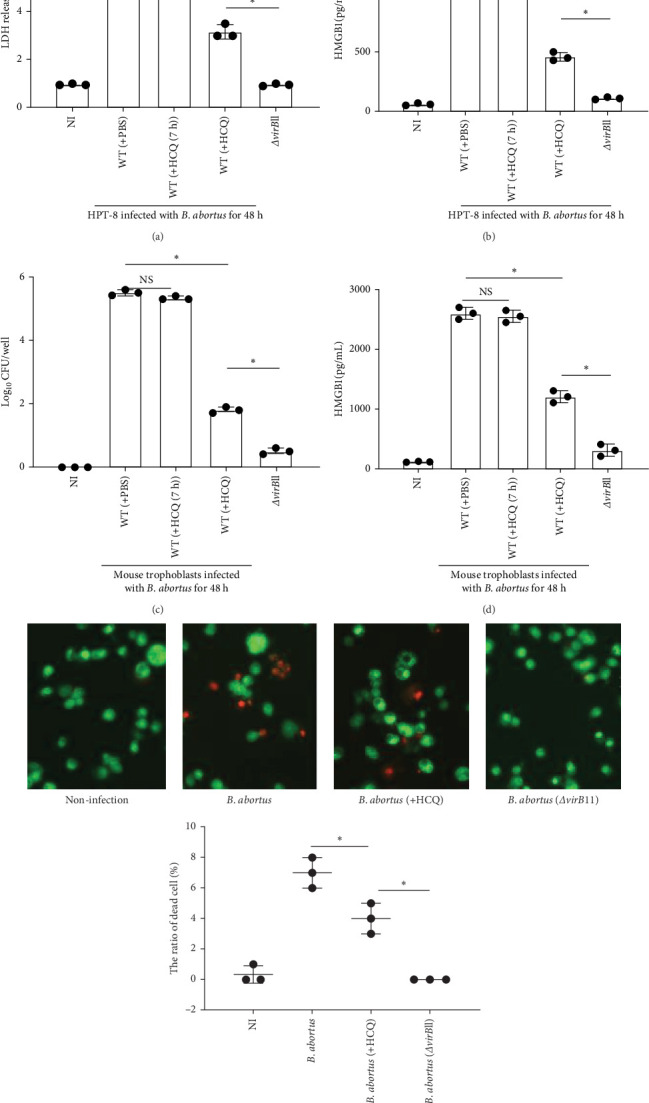
HCQ can reduce trophoblasts death HCQ reduces trophoblasts death through inhibiting *Brucella* intracellular growth. Mouse trophoblasts or HPT-8 cells were infected with WT or (MOI = 100) in the presence of PBS, HCQ, or (HCQ (7 h) (1 μg/mL) for 48 h. *ΔvirB*11 *B. abortus* infection (MOI = 100) was served as a positive control. (A) The levels of LDH in the supernatants of HPT-8 cells were analyzed by ELISA. (B) The levels of HMGB1 in the supernatants of HPT-8 cells were analyzed by ELISA. (C) The bacterial number within mouse trophoblasts was measured by CFU assay. (D) The levels of HMGB1 in the supernatants of mouse trophoblasts were analyzed by ELISA. (E) *B. abortus*-infected mouse trophoblasts were stained with PI (propidium iodide). The necrotic cells are PI-positive (red) (The scale is 75 μm). One-way ANOVA followed by Bonferroni correction is used for analyzing the results. *p*-Values less than 0.05 is statistically significant (*⁣*^*∗*^, *p<*0.05). NS: no significance.

It was reported that CQ can reduce trophoblast cell death by inhibiting chaperone-mediated autophagy in trophoblast cells [27]. Next, we investigated whether HCQ also can reduce trophoblasts death via inhibiting bacterial intracellular growth. HPT-8 cells were infected with *B. abortus*, treated with antibiotics to kill extracellular bacteria, and cultured supernatants were harvested at 48 h pi to determine the role of HCQ in mediating the viability of cells. *ΔvirB*11 *B. abortus*-infected cells was designed as positive control. The levels of LDH release were significantly inhibited in the presence of HCQ compared to nontreatment or HCQ (7 h) treatment group (Figure 4A). Since trophoblasts death caused by *Brucella* infection can lead to release of high mobility group box 1 (HMGB1) into the extracellular media [7], the supernatants were also collected to determine the release of HMGB1 into the media. HCQ treatment significantly inhibited the release of HMGB1 compared to nontreatment or HCQ (7 h) treatment group (Figure 4B). To confirm the role of HCQ in inhibiting cell death, primary mouse trophoblasts were utilized to infected with *B. abortus*. In line with HPT-8 cells, HCQ significantly suppressed bacterial intracellular replication (Figure 4C) and the release of HMGB1 into media of mice trophoblasts (Figure 4D), but late HCQ treatment resulted in similar intracellular CFU numbers and HMGB1 release as nontreatment (Figure 4C,D). We also evaluated trophoblast cell death by Propidium iodide (PI)-staining. HCQ treatment significantly decreased the ratio of dead cells compared to untreated cells (Figure 4E). Taken together, these data suggest that administration of HCQ can reduce trophoblasts death through inhibiting *Brucella* intracellular growth.

### 3.5. HCQ Inhibits Secretion of Inflammatory Cytokines Caused by *Brucella*-Infected Trophoblasts

**Figure 5:**
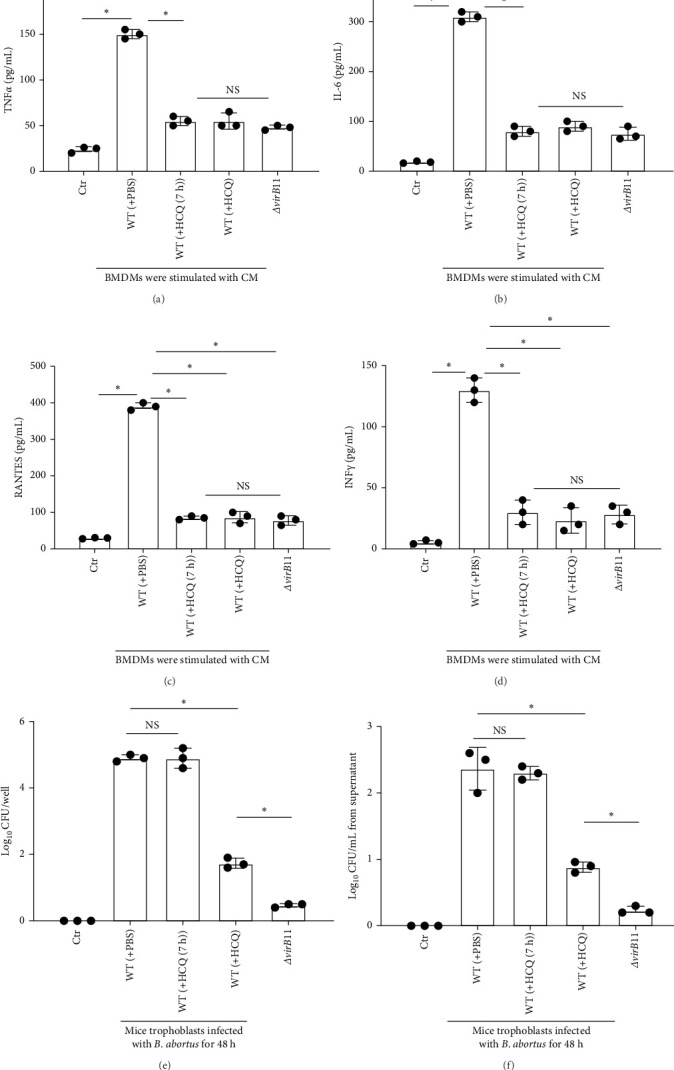
Administration of HCQ inhibits the secretion of inflammatory cytokines caused by *Brucella*-infected trophoblasts. BMDMs were stimulated with conditioned media (CM) from *B. abortus* -infected mouse trophoblasts which was treated with PBS, HCQ, or HCQ (7 h) (1 μg/mL). CM from uninfected mouse trophoblasts was served as negative control. CM from *ΔvirB*11 *B. abortus* -infected mouse trophoblasts was served as a positive control. After 24 h treatment, productions of TNFα (A), IL-6 (B), RANTES (C), and IFN-γ (D) in the media of BMDMs were measured by ELISA. (E) The bacterial number within trophoblasts was measured by CFU assay. (F) The number of extracellular bacteria. One-way ANOVA followed by Bonferroni correction is used for analyzing the results. *p*-Values less than 0.05 is statistically significant (*⁣*^*∗*^, *p<*0.05). NS: no significance.

*B. abortus* infection led to death of placental trophoblasts and release of danger-associated molecular patterns (DAMPs) and pathogen-associated molecular patterns (PAMPs) into the extracellular milieu that activate Toll-like receptors (TLRs) to produce inflammatory cytokines [6]. Considering that HCQ can inhibits the effects of TLRs [16], we determined whether HCQ is involved in inhibition of production of inflammatory cytokines, such as IL-6, TNF, RANTES, and IFN-γ, which play essential roles in the pathophysiology of placentitis [9, 10, 12, 28]. To this aim, bone marrow-derived macrophages (BMDMs) were treated with CM from the *B. abortus*- infected mouse trophoblasts. Cytokines were analyzed in the BMDMs' supernatants at 24 h post treatment. Levels of TNFα, IL-6, RANTES, and IFN-γ already present in the CM were subtracted to analyze the cytokines' levels triggered by treatment. The stimulation of BMDMs with CM, in which the infected trophoblasts were treated by PBS, resulted in a significant increase in TNFα (Figure 5A), IL-6(Figure 5B), RANTES (Figure 5C), and IFN-γ (Figure 5D) production compared to BMDMs stimulated with CM, in which infected mouse trophoblasts were treated by HCQ. Moreover, we did not observe obvious differences of cytokines production between stimulation with CM from containing HCQ and CM from *ΔvirB*11 *B. abortus*-infected cells. Moreover, HCQ (7 h) treatment leads to similar number of intracellular (Figure 5E), extracellular bacteria (Figure 5F), or HMGB1 release (one of DAMPs), which is T4SS dependent (Figure 4D), as PBS treatment. These results suggest that that HCQ might inhibit secretion of cytokines caused by *Brucella* -infected trophoblasts, which is T4SS independent.

### 3.6. HCQ Inhibits Secretion of Inflammatory Cytokines via Inactivation of mitogen-activated protein kinases (MAPKs) and NF-κB

**Figure 6:**
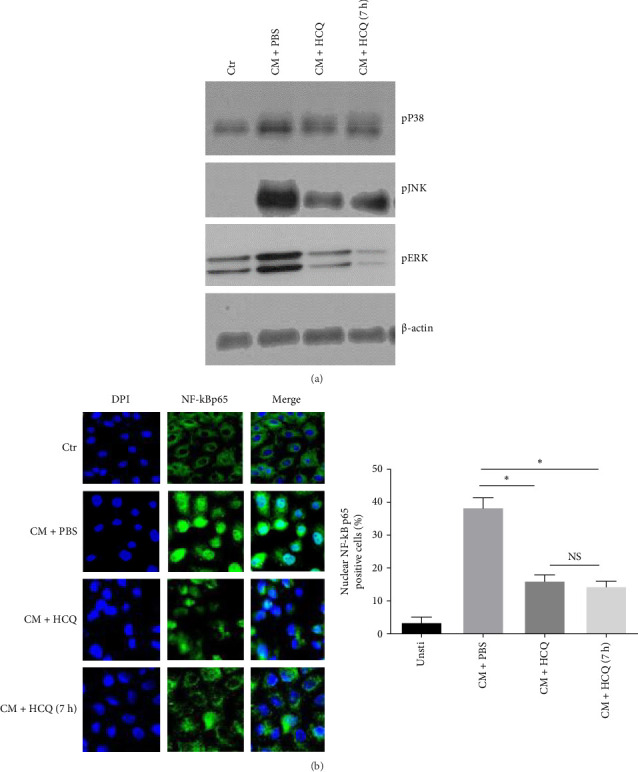
HCQ inhibits the secretion of inflammatory cytokines via the inactivation of MAPKs and NF-κB. BMDMs were stimulated with CM from *B. abortus* -infected mouse trophoblasts which was treated with PBS, HCQ or HCQ (7 h) (1 μg/mL). CM from uninfected mouse trophoblasts was served as control. (A) After 24 h stimulation, phosphorylated, and total ERK, p38 and JNK in BMDM were analyzed by western blotting. (B) The nuclear translocation of NF-κB in BMDM is analyzed by immunostaining image. NF-κB subunit p65: green and DPI: blue (the scale is 10 μm). One-way ANOVA followed by Bonferroni correction is used for analyzing the results. *p*-Values less than 0.05 is statistically significant (*⁣*^*∗*^, *p<*0.05). NS: no significance.

Among the most proximal events, in which DAMPs or elements of *Brucella* modulates production of inflammatory cytokines in BMDM, is the activation of MAPKs [7]. To test if HCQ treatment affected the activation of MAPK, phosphorylation of p38, JNK, and ERK in BMDM were analyzed by western blotting. After stimulation with CM from *B. abortus* -infected mouse trophoblasts for 24 h, phosphorylation of p38, ERK, and JNK were increased in BMDMs. In contrast, phosphorylation of p38, JNK, and ERK in BMDMs were decreased when CM from HCQ or HCQ (7 h)-treated *B. abortus* -infected cells was used for stimulation (Figure 6A). This data suggests that HCQ works as an upstream inhibitor of MAPK activation. Since NF-κB activation has been shown to play an essential role in regulation of inflammatory cytokines production [29], we also investigated the role of HCQ in CM mediated NF-κB inactivation in BMDMs by confocal microscope (Figure 6B). CM from HCQ treatment (HCQ or HCQ [7 h]) decreased nuclear translocation of NF-κB p65 of trophoblasts compared to CM from *B. abortus* -infected cells. Collectively, these results indicate that HCQ suppresses inflammatory signaling pathways caused by *B. abortus* infection.

### 3.7. HCQ Suppresses the Production of Inflammatory Cytokines in Mice Placental Explants Incubated With *Brucella* Ex Vivo

**Figure 7:**
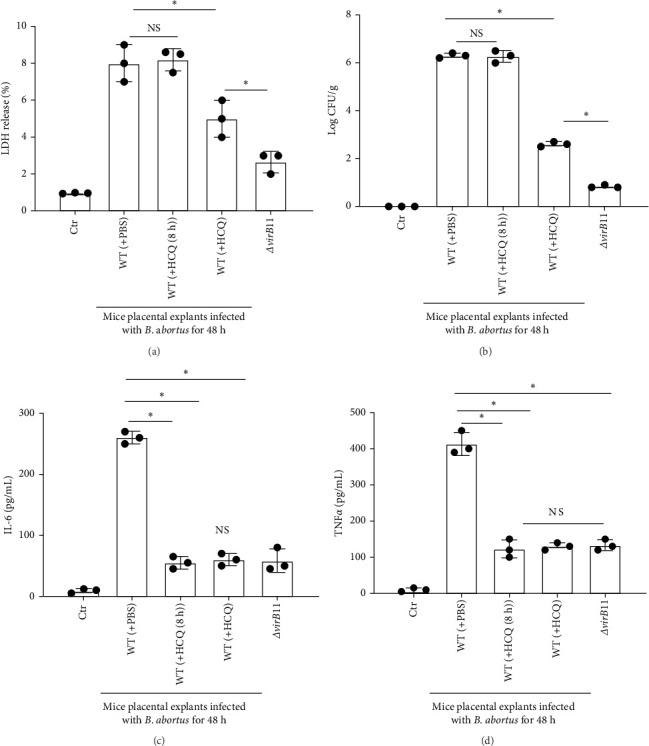
HCQ suppresses cytokine secretion in mouse placental explants infected with *B. abortus* ex vivo. Mouse explants were incubated with WT *B. abortus* in the presence of PBS or HCQ (1 μg/mL) or HCQ (1 μg/mL) HCQ (1 μg/mL) treatment after 8 h of *Brucella* infection (HCQ [8 h]). Uninfected mouse explants were served as a negative control (Ctr). *ΔvirB*11 *B. abortus* -infected mouse explants were served as a positive control. (A) After 48 h *Brucella* incubation, LDH release was measured using commercially obtained assay kits. (B) The number of *Brucella* within mouse explants was analyzed by CFU assay. Culture media of mouse explants were collected to measure IL-6 (C) and TNFα (D) secretion through ELISA. One-way ANOVA followed by Bonferroni correction is used for analyzing the results. *p*-Values less than 0.05 is statistically significant (*⁣*^*∗*^, *p<*0.05). NS: no significance.

The above results suggest that HCQ treatment not only inhibits trophoblasts death caused by *Brucella* infection but also the secretion of cytokines by macrophages. To investigate the global function of HCQ in the mouse placenta, in which cells interactions in placenta may occur in the context of the natural tissue architecture and cellular proportions, explants from mouse placenta were infected with WT or *ΔvirB*11 *B. abortus* for 48 h in the absence or presence of HCQ. Like *B. abortus*-infected trophoblasts, HCQ treatment reduced the release of LDH compared to untreated explants or HCQ (8 h) treatment (HCQ treatment after 8 h of *Brucella* infection) (Figure 7A). Consistent with LDH release, HCQ treatment suppressed bacterial growth within explants (Figure 7B). Although we did not observe the obvious difference of bacterial intracellular growth between PBS and HCQ (8 h) treatment (Figure 7B), both HCQ or HCQ (8 h) treatment significantly decreased the production of IL-6(Figure 7C) and TNFα (Figure 7D).

### 3.8. Administration of HCQ Inhibits Abortion and Placentitis Caused by *B. abortus* Infection in Mice

**Figure 8:**
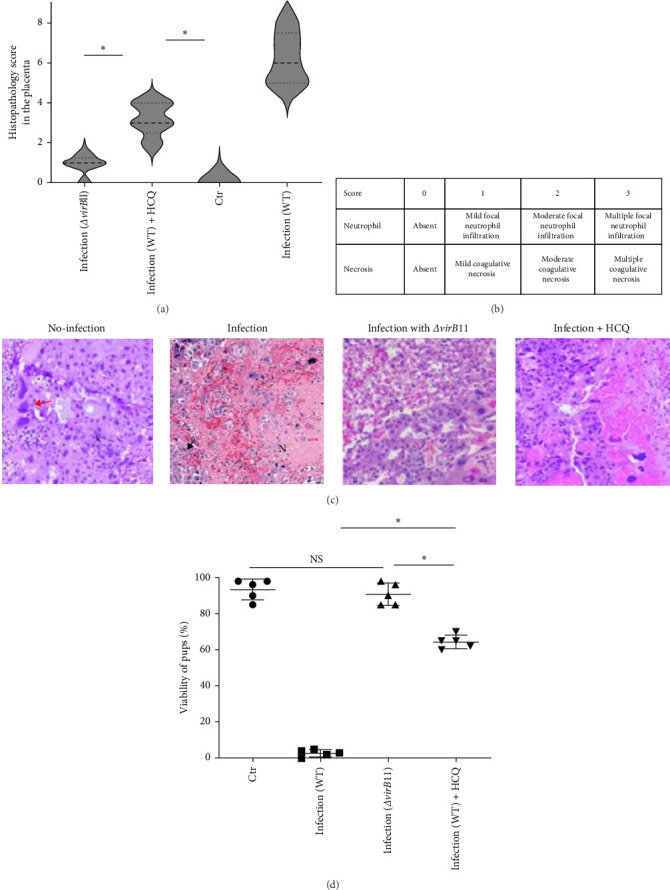
Administration of HCQ inhibits *B. abortus* infection-induced mice abortion and placentitis. The 10 pregnant mice were infected i.p. with WT *B. abortus*. The five pregnant mice were infected i.p. with *ΔvirB*11 *B. abortus*. The five uninfected pregnant mice were used as control (Ctr). For WT *B. abortus*-infected pregnant, half of mice were treated with HCQ. (A) Histopathology analysis at 15 days postinfection (the scale is 100 μm). (B) The scoring standard for evaluating hematoxylin and eosin (H&E) − stained sections from *B. abortus*–infected mice placenta. (C) Representative histopathological images of placentas. N indicates cell death. Neutrophil infiltration was shown by black arrow. Trophoblast was shown by red arrow. (D) Viability of mouse pups at 15 days postinfection, was measured through checking the presence of heartbeat and fetal movement. Each dot represents one animal. One-way ANOVA followed by Bonferroni correction is used for analyzing the results. *p*-Values less than 0.05 is statistically significant (*⁣*^*∗*^, *p<*0.05). NS: no significance.

*B. abortus* infection can result in chronic mild inflammation during its persistence within its natural goat or sheep reservoir or within mice. However, *B. abortus* causes severe acute inflammation in the placenta of pregnant mice or goats, which leads to abortion [6]. Building on the above data, we then investigated whether HCQ could inhibit acute placentitis in pregnant mice infected with *B. abortus* [10]. The pregnant mice were treated i.p. at days 3, 5, and 9 post infection with HCQ. Because T4SS deficient *Brucella* infection fails to induce trophoblast necrosis and abortion [12, 13], *ΔvirB*11 *B. abortus*-infected pregnant mice were included as the positive control group. When *B. abortus*-infected pregnant mice were treated with HCQ, the histopathologic severity of placentitis was significantly decreased compared to the untreated infected mice (Figure 8A–C). Administration of HCQ to infected pregnant mice leads to a significant increase in pup viability compared to nontreatment. Pup viability of infected pregnant mice was <6% in control group but improved significantly to 65% with HCQ treatment (Figure 8D). These data suggest the HCQ treatment significantly inhibits abortion caused by *B. abortus* infection.

## 4. Discussion

In this study, we provided the first evidence that the antimalarial drug HCQ, extensively used in the treatment of many rheumatic diseases, such as SLE and RA, suppresses abortion caused by *B. abortus* infection. Mechanistically, we found that administration of HCQ raises the pH value of eBCV and then downregulates T4SS expression of intracellular *Brucella*, resulting in decreased *Brucella* replication within trophoblasts and decreased trophoblast cell death. Moreover, HCQ inhibits secretion of inflammatory cytokines via inactivation of MAPKs and NF-κB. Although the use of HCQ has a good safety record and improves pregnancy outcomes in SLE [15], our study provides a causative link between HCQ and an antiabortion drug. Based on our current knowledge about the pathophysiology of *Brucella* infection-induced abortion, further clinical investigations on the potential benefit of HCQ in abortion are expected.

In this study, we further report that HCQ inhibits placentitis and reduces abortion caused by *B. abortus* infection. Generally, the main virulence factor required for abortion in the mouse is the *Brucella* T4SS, which is encoded by *Brucella virB*1–12 operon. The T4SS can inject some effectors into trophoblasts, resulting in severe ER stress driving cellular necrosis. The dead trophoblasts release DAMPs, driving TLR-triggered acute inflammatory response of the placenta, resulting in the death of mouse pups. We show that HCQ reduces abortion in infected mice in both T4SS-dependent and independent manner. Namely, HCQ function to alleviate bacterial replication within trophoblasts and cell death in T4SS-dependent manner; administration of HCQ suppresses acute inflammation of the placenta in T4SS-independent manner. This novel mechanism by which HCQ reduce *B*. *abortus* infection-induced abortion is the major discovery of this study and should lead to larger animal model studies.

Another important finding is that inflammation plays an essential role in inducing abortion caused by *B*. *abortus* infection. Our previous study demonstrated that pup viability of infected pregnant mice was improved significantly to 30% with anti-HMGB1 treatment [7], but here we find that HCQ improves pup viability to 65%. A careful comparison of the two models of treatment reveals some intrinsic differences. HCQ can inhibit IL-6, TNFα, INFγ, and RANTES, whereas neutralizing HMGB1 only inhibited the production of IL-6 and TNFα. Similarly, progesterone [8], or anti-TNFα antibody treatment, does not result in a similar inhibition of inflammation as HCQ treatment. It is notable that HCQ treatment for *B. abortus*-infected pregnant mice has the same inhibition of inflammatory cytokines as *ΔvirB*11 *B. abortus*-infection, while not providing complete prevention of abortion as *ΔvirB*11 *B. abortus*. This discrepancy could be interpreted by the fact tha*t ΔvirB*11 *B. abortus* could not secret effectors into trophoblasts, whereas HCQ treatment just decreased the secretion of the T4SS effector (Figure 2C). This speculation is supported by the following evidence: *ΔVceC B. abortus* infection improves pup viability of infected mice compared to WT infection [10, 13].

Early acidification of eBCV is essential for intracellular growth in host cells [22, 24, 25]. Because acidification of eBCV during the early stage of infection can be prevented by treatment of host cells with monensin or bafilomycin, they were often utilized to investigate the mechanism of *Brucella* intracellular replication. However, they were cytotoxic to host cells. HCQ, as a weak base, alkalinizes macrophage phagolysosome and has no cytotoxicity to host cells [16]. Moreover, administration of HCQ can raise the pH value of eBCV, and suppress multiplication of *B. abortus* within trophoblasts via decreasing T4SS expression, which is in line with the effect of treatment of bafilomycin or monensin. We think that HCQ also can be used as a “new” drug to study the *Brucella* infectious mechanism.

## Data Availability

The data that support the findings of this study are available from the corresponding author upon reasonable request.

## Supporting Information

Supporting Information Supporting Information section provides the detailed description of the Materials and Methods.
