# Supplementary material for: Effect of Hydroxychloroquine in a Mouse Model of Abortion Caused by Brucella abortus Infection
Source: Transbound Emerg Dis. 2025 Aug 8;2025:8853857. doi: 10.1155/tbed/8853857 (PMC12356681; doi:10.1155/tbed/8853857)
Supplement: Supporting Information — section provides the detailed description of the Materials and Methods. [file 8853857.f1.docx]

**Supplementary information**

**Ethics Statement**

The animal protocol was carried out following the regulation of Jilin University Animal Care and Use Committee. The related animal procedures also comply with the Guide for the Care and Use of laboratory Animals (NIH Publication No. 85-23, revised 1996). The study has been reported in accordance with ARRIVE guidelines and is approved by the animal experimental ethical committee of Jilin University (Agreement Number: KT202408159).

**Materials**

Anti-RANTES (Cat# 36467), p38 (Cat#9212), pho-P38(Cat#4511), ERK(Cat#4695), pho-ERK(Cat#4370), JNK(Cat#9252) and pho-JNK(Cat#4668) were purchased from CST (Cell Signaling Technology) (Shang Hai, China). Anti-β-actin (Cat#sc-69879) was from Santa Cruz Biotechnology (Shang Hai, China). HCQ(Cat#509272) and Other chemicals were purchased from Sigma-Aldrich (Shang Hai, China) if they were not indicated in the paper. Drug concentrations in the working buffer are expressed either as the final molar or μg/ml concentration.

**Bacterial strains and growth conditions**

*B. abortus* 2308 strain (WT) or Δ*virB*11 *B. abortus* was a kind gift from Dr. Ding’s laboratory (China Institute of Veterinary Drug Control, Beijing, China) or Dr. Chen’s laboratory (Institute of Disease Control and Prevention, AMMS Beijing, China), respectively. *B. abortus* was grown either on TSA (tryptic soy agar plates) or in TSB (tryptic soy broth). The numbers of *B. abortus* in cultures were calculated by comparing the OD at 600 nm (OD600) with a standard curve, whereas the actual concentration of inoculation was numerated by plating on TSA plates. All live *B. abortus* manipulations were performed in BSL3 (Biosafety Level 3) facilities based on standard operating protocols approved by the Biosafety Committee of Jilin University.

**Growth curve in TSB**

*Brucella* growth was measured at an optical density at 600 nm (OD600). For growth curve analysis, bacterial strains were incubated in TSB for 24 h, and then diluted with TSB to an OD600 value of 0.01 and cultured in a rotary shaker (200 rpm) at 37 °C in the presence or absence of HCQ (1 or 10μg/ml). Cultures were taken at appropriate interval and OD600 values were recorded.

**Mice bone marrow derived macrophages (BMDMs) culture**

BMDMs were purified and cultured as previously described[1]. Briefly, BMDMs were derived from flushed bone marrow from mouse long bones. After 2 days, non-adherent cells were collected and transferred at a density of 5 × 10^6^ cells and were cultured for an additional 2 to 5 days. BMDMs were maintained in αMEM (Gibco, Cat#12571063) supplemented with 10% endotoxin-free fetal bovine serum (Gibco, Cat#A5670701), 5% CMG (conditioned medium containing M-CSF), penicillin, streptomycin, and glutamine. The purity of BMDMs were assessed by flow cytometry, and had more than 95% CD11b^+^-positive cells.

**Isolation of mice placenta**

The mice placenta was isolated from pregnant mice following previously described procedures[2]. Briefly, at 10 days of gestation, the uterus was incised from the pregnant mice to expose placenta. Each fetus was removed with a hemostatic clamp by pinching its umbilical cord after the fetal wraps were torn. Then, each placenta was taken and put in a sterile container. The placentas were kept at 4°C for no more than 24 h in DMEM (Gibco, Cat#11965092) complete tissue culture medium and transported to the lab for processing.

**Mice trophoblasts isolation**

Mice trophoblasts were isolated from placenta of the pregnant mice following previously described procedures [2]. Briefly, at 10 days of gestation, coarsely minced placental tissue was washed thoroughly with PBS and incubated with buffer containing collagenase and trypsin at 37°C for 30 min. Dissociated tissue was passed through a cell strainer to take undigested materials. After sedimentation and centrifugation, the supernatant was obtained and mixed with washing media containing FCS (fetal calf serum) to cease enzymatic digestion. Then the samples were centrifuged, and the pellet of sample was treated with collagenase for about 3 minutes. After several centrifugations and washes the cell pellet was re-suspended with DMEM complete culture medium and plated in multi-well plates at 37°C and 5% CO_2_. Following this procedure, the murine trophoblastic cells isolated from term placenta of murine can survive with DMEM complete culture medium for 7 days. The isolated murine trophoblasts also are used for *Brucella* infection directly. The purity of the murine trophoblasts is determined through immunofluorescence with an anti-cytokeratin-7 antibody (BD, Cat# 550507). All the cell suspensions analyzed had more than 95% cytokeratin 7-positive cells[3].

**Administration of HCQ during *Brucella* infection**

C57BL/6 mice (8–10 weeks) were held in a BSL3 (biosafety level 3) laboratory. Male mice were mated with female mice. The presence of a vaginal plug in the female mouse was selected as pregnant mouse. The groups of four to six pregnant mice were infected intraperitoneal (i.p.) with 1× 10^5^ CFU of *B. abortus* at 5 days of gestation. The infected pregnant mice were treated i.p. at days 3, 5, 9 post-infection with HCQ (100 mg/Kg). Mice were euthanized after 12 days of treatment (corresponding to day 17 of gestation). The placentas of mice were collected aseptically. Percentage of viability was calculated as.

Pup viability was evaluated based on the presence of fetal movement and heartbeat and fetal size and skin color as previously described[4]. According to Gounder’s method[4], percentage of viability was calculated [(number viable pups per litter/total number pups per litter) ×100]. Meanwhile, these placenta samples were collected for histopathological analysis.

**Histopathology**

Mice placentas were fixed with formalin and embedded in paraffin, and then the samples were cut into 6-um thick sections. Placenta tissues were stained with HE (hematoxylin-eosin). Slides were analyzed for tissue damage and placentitis. The neutrophils and necrotic area (×100) were assessed by a veterinary pathologist in a blind manner (n=5)[5]. For the observation of treatment group, the sections were chosen by random[2, 5].

**HPT-8 or mice trophoblasts infection and survival assay**

HPT-8 (a trophoblast cell line) (2.0×10^5^), were cultured in αMEM with 10% fetal bovine serum (FBS) at 37 °C with 5% CO_2_. Primary mice trophoblasts (1.0×10^4^) were plated in 24-well plates without antibiotics in complete tissue culture media and incubated overnight with 5% CO_2_ at 37°C. And then, trophoblasts were infected with *B. abortus* at MOI (multiplicity of infection) of 100:1 by centrifuging *B. abortus* into cells at 400g at 4°C for 10 min. Following 15 min of infection in an atmosphere containing 5% CO2 at 37°C, αMEM medium was used to wash the cells three times to take off extracellular *B. abortus* and infected for another 60 min in αMEM with 40μg/ml gentamicin to kill extracellular *Brucella*. Thereafter, the antibiotic concentrations were decreased to 10 μg/ml[6]. To investigate *Brucella* intracellular growth, infected HPT-8 cells were lysed with PBS with 0.1% Triton X-100 at indicated time points, and the serial dilutions of lysates were plated immediately into TSA plates to count CFUs [2, 6].

**Mice placental explants culture and infection**

The mice placentas were obtained at 10 days of gestation. Mice placental explants were obtained after the removal of the extra-chorionic membranes and marginal hematoma. Then, the explants were cultured in 24 –well plate in DMEM medium (containing 10% FBS) without antibiotics. To infect the explants, 1x10^8^ CFU of *B. abortus* were added to each well by centrifuging *B. abortus* into cells at 400g at 4°C for 10 min. Following 15 min of infection in an atmosphere containing 5% CO_2_ at 37°C, PBS was used to wash the explants three times to take off extracellular *B. abortus* and infected for another 60 min in DMEM with 40μg/ml gentamicin to kill extracellular *Brucella*. At 48 hour post infection (h.p.i), the supernatants of explants were collected to measure LDH release and cytokines. To investigate *Brucella* intracellular growth, explants homogenates were manually performed in 1 ml of sterile PBS using a tissue homogenizer and were plated on TSA to count CFU[2].

**Measurements of cytokine in culture supernatants**

The level of RANTES (Fine Test, Cat# EM0164 ), INFγ (ThermoFisher, Cat # PA5-97783), TNF(ThermoFisher, Cat # BMS607-3) and IL-6 (ThermoFisher, Cat # 88-7064-88)were analyzed in culture supernatants from *B. abortus*-infected explants or cells by ELISA kits based on the manufacturer’s instructions.

**LDH (Lactate dehydrogenase) assay**

The concentration of LDH released from the cells or placental explants was evaluated by a commercial kit of LDH assay (abcam, Cat #ab102526) based on the manufacture’s procedure. In brief, supernatants of cells or placental explants were collected at indicated time points, the kit reagent was added into an aliquot of supernatant (50 μl) and incubated for 45 min, and then the reaction was halted and the absorbance analyzed at 560 nm through the reader of an ELISA plate. Released LDH from the trophoblasts or placental explants was expressed as a percentage of total LDH activity. Total LDH was determined by lysing the cultures with Triton X-100[2].

**Western blot**

Cells were lysed in ice-cold RIPA (radio-immunoprecipitation) mild detergent buffer. The protein content of cells was analyzed by the kit of BCA protein evaluation (ThermoFisher, Cat # A65453). About 20 mg protein of lysates was loaded to SDS–PAGE and then transferred to PVDF membrane. Membrane was incubated with primary antibody at 4°C overnight, and then washed with PBS three times at room temperature, followed by incubation with suitable secondary antibody for 2 hours at room temperature. Protein bands of membrane were shown by the reagent of ECL (GE Healthcare, Cat #RPN68D)[7].

**Immunofluorescence Staining**

HPT-8 cells were fixed with 1.5% paraformaldehyde for 2 hours at room temperature (RT). Then, the samples were blocked with 2.4G2 antibody (CST, Cat#80366S) for 1 hour at RT. The samples were incubated with appropriate first antibody at 4°C overnight. cells were washed with PBS and incubated with secondary fluorescent antibody for 1 hour at RT. The immunofluorescence staining was observed under confocal microscope (Zeiss LSM510). The images were collected through the software of LSM confocal microscope.

References

1. Ren J, Hou H, Zhao W, Wang J, Peng Q: **Administration of Exogenous Progesterone Protects Against Brucella abortus Infection-Induced Inflammation in Pregnant Mice**. *J Infect Dis* 2021, **224**(3):532-543.

2. Liu X, Zhou M, Wu J, Wang J, Peng Q: **HMGB1 release from trophoblasts contributes to inflammation during Brucella melitensis infection**. *Cell Microbiol* 2019, **21**(10):e13080.

3. Pennington KA, Schlitt JM, Schulz LC: **Isolation of primary mouse trophoblast cells and trophoblast invasion assay**. *J Vis Exp* 2012(59):e3202.

4. Keestra-Gounder AM, Byndloss MX, Seyffert N, Young BM, Chavez-Arroyo A, Tsai AY, Cevallos SA, Winter MG, Pham OH, Tiffany CR *et al*: **NOD1 and NOD2 signalling links ER stress with inflammation**. *Nature* 2016, **532**(7599):394-397.

5. Tsai AY, Byndloss MX, Seyffert N, Winter MG, Young BM, Tsolis RM: **Tumor Necrosis Factor Alpha Contributes to Inflammatory Pathology in the Placenta during Brucella abortus Infection**. *Infect Immun* 2022, **90**(3):e0001322.

6. Celli J, de Chastellier C, Franchini DM, Pizarro-Cerda J, Moreno E, Gorvel JP: **Brucella evades macrophage killing via VirB-dependent sustained interactions with the endoplasmic reticulum**. *J Exp Med* 2003, **198**(4):545-556.

7. Li C, Wang J, Sun W, Liu X, Wang J, Peng Q: **The Brucella Effector BspI Suppresses Inflammation via Inhibition of IRE1 Kinase Activity during Brucella Infection**. *J Immunol* 2022, **209**(3):488-497.
